# Nonlinear recurrence analysis of piezo sensor placement for unmanned aerial vehicle motor failure diagnosis

**DOI:** 10.1038/s41598-024-58606-6

**Published:** 2024-04-09

**Authors:** Andrzej Koszewnik, Leszek Ambroziak, Daniel Ołdziej, Paweł Dzienis, Bartłomiej Ambrożkiewicz, Arkadiusz Syta, Ghada Bouattour, Olfa Kanoun

**Affiliations:** 1 Bialystok University of Technology, Wiejska Street 45C, Bialystok, 15-351 Poland; 2 Lublin University of Technology, Nadbystrzycka 38, Lublin, 20-618 Poland; 3 Leuphana University Luneburg, Universitätsallee 1, Lüneburg, 21335 Germany; 4 Chemnitz University of Technology, Reichenhainer Strasse 70, Chemnitz, 09111 Germany

**Keywords:** Unmanned aerial vehicle, Propulsion system, Daignostic piezo sensor, Nonlinear dynamics analysis, Reccurence anslysis, Aerospace engineering, Mechanical engineering

## Abstract

This paper is focused on the diagnostics of multicopter UAV propulsion system, in which the temporary transient states occur during operation in faulty conditions (eg. not all motor phases working properly). As a diagnostic sensor, the piezo strip has been used, which is very sensitive to any vibrations of the multi-rotor frame. The paper concerns the precise location of the sensor for more effective monitoring of the propulsion system state. For this purpose, a nonlinear analysis of the vibration times series was carefully presented. The obtained non-linear time series were studied with the recurrence analysis in short time windows, which were sensitive to changes in Unmanned Aerial Vehicle motor speeds. The tests were carried out with different percentage of the pulse width modulation signal used for the operation of the brushless motor and for different locations of the piezosensor (side and top planes of the multicopter arm). In the article, it was shown that the side location of the piezosensor is more sensitive to changes in the Unmanned Aerial Vehicle propulsion system, which was studied with the Principal Component Analysis method applied for four main recurrence quantifications. The research presented proves the possibility of using nonlinear recurrence analysis for propulsion system diagnostics and helps to determine the optimal sensor location for more effective health monitoring of multicopter motor.

## Introduction

Unmanned Aerial Systems (UAS) have gained immense importance in various inaccessible domains such as agricultural inspection, infrastructure assessment (e.g., train rails), energy source monitoring (e.g. wind turbines, solar cells) and many others^1–6^. A typical UAS consists of a drone, also known as an Unmanned Aerial Vehicle (UAV), along with a Ground Control Station (GCS), a command and control link (C2 link), a human UAV Operator (UAVO), and various types of equipment such as specialized sensors, cameras and other types of payloads. UAVs often operate in demanding environments that include harsh weather conditions (such as strong winds, gusts, high humidity, and rain) and the potential for accidents, including propulsion unit failures^7,8^. Therefore, it is crucial to have control mechanisms in place to monitor the working conditions and state of the UAV, considering factors such as aging and damage. Faults can impact various aspects of UAV operation, such as power supply, communication, and the operation of motors, frames, and sensors.

In fixed-wing UAVs, it is possible to glide and perform a controlled landing even if the engine fails or there is a fault in another part of the system (eg. mechanism of control surfaces). However, in the case of multicopters, which are the second most common type of UAVs, there is no possibility of gliding or performing an emergency autorotation due to their mechanical structure. This means that monitoring the state of UAVs becomes even more critical, particularly for Vertical Take-Off Landing (VTOL) UAVs. In this category of UAVs, a failure of the propulsion system can result in complete destruction of the vehicle^9^. Motor failure detection in UAVs can be achieved by using various indicators such as temperature, sound level, and vibration measured by gyroscopes or acceleration sensors. Some systems employ dual sensors to increase the sensitivity to failure^10–14^. However, the inclusion of these sensors typically requires additional electronic circuits, resulting in increased weight and power consumption, particularly when they are associated with each arm of the drone. Furthermore, the selection of the accelerometer sensor to achieve optimal reliability in real-time fault detection requires further in-depth investigation^15^. The placement of sensors presents its own challenges, as they can be located on the side of the arm, on top of the arm, or on the propeller^16^. Alternatively, to minimize the reliance on sensors within the UAV, the signal processing system and the acceleration signal taken from the Inertial Measurement Unit (IMU) sensors can be used as a solution to detect failure without the need to mount additional sensors^17^. Another proposed approach involves the use of a single accelerometer integrated with the drone to detect failures^18,19^. However, this system cannot identify the specific source and direction of failure. To detect motor failures, the use of machine learning approaches is commonly adopted, which involve data collection and implementation. Although machine learning can help identify failures, it does not guarantee proper control of the UAV to ensure stable flight. Therefore, it is necessary to thoroughly study and evaluate an efficient machine learning model in terms of proper algorithm implementation (supervised or unsupervised)^20^, as well as selection of relevant features. It should be noted that while the machine learning concept helps identify failures, it does not address the issue of controlling the UAV when the selected sensors and extracted features are inadequate.

To address these challenges, several studies^21–27^ have explored the analysis of the location of piezoelectric sensors using the finite element method (FEM). The objective of these studies is to detect faults using a lightweight sensor system and a streamlined methodology for UAVs, reducing the time required to reconfigure the control law and minimize the effects of crashes. Another instance in this field is a paper^28^ where different types of piezo sensors are located on the frame UAV to harvest energy from vibrating structure of the UAV . Specifically, the investigation focuses on fault detection using piezo sensors placed on the arms of the UAV frame.

During the investigations presented in this paper, time series and frequency domain analyzes are conducted through the measurement and recording of vibration data. Nonlinear data processing methods such as recurrence plots (RP), recurrence quantification analysis (RQA), and principal component analysis (PCA) are applied to evaluate the effect of each piezo-patch sensor on the detection of anomalies in motor operation. Additionally, the investigation takes into account the varying duty cycles of the PWM motor control signal. The analysis conducted enables accurate placement of the sensor deployment on the multicopter frame to efficiently detect failures.

This paper is organized as follows: “Project concept and preliminary investigations” section presents a simulation of the homogeneous model of a macrofiber composite (MFC) located on the multicopter arm. The purpose of this part of the study was to obtain an initial characteristic of the frequency response for the analysis of the intact and damaged UAV motor arm as well as the study of piezosensor placement. In “Piezoelectric sensor location on the UAV arm” section experimental investigations were carried out for the intact and damaged propulsion system for the chosen value of the duty cycle of the PWM signal. The recorded voltage output signals from both piezosensors indicated that one of them allows faster detection of faults in the propulsion system.

In “Analysis on the amount of piezoelectric sensors” section describes the process of selection of feature for fault diagnosis using recurrence plots (RP) and recurrence quantification analysis (RQA) methods. In Additionially, it the same section the principal component analysis for the UAV arm structure with the intact and damaged propulsion system is described. In the last Section the main findings of this work were concluded.

## Project concept and preliminary investigations

### Project concept

Proper location of piezosensors on the multicopter arm structure allows faster detection of a propulsion system fault. The aim of the project is to study the amount and location of piezoelectric sensors for the detection of failure. The study of the number and position of sensors (on the top, on the side or both locations) is elaborated by the analysis of various feature selection techniques using nonlinear analysis time series methods like Recurrence Plots (*RP*), Recurrence Quantification Analysis (*RQA*), statistical method by Principal Component Analysis (*PCA*), and frequency domain analysis. This investigation has been carried out for different BLDC motor duty cycles.

The position of the sensors is elaborated on the basis of Finite Element Method (FEM) simulation regarding the possible forces generated by the motor and propeller located at the end of the UAV arm. These investigations help define the optimal piezoelectric location for more efficient detection of failure in the UAV.

**Figure 1:**
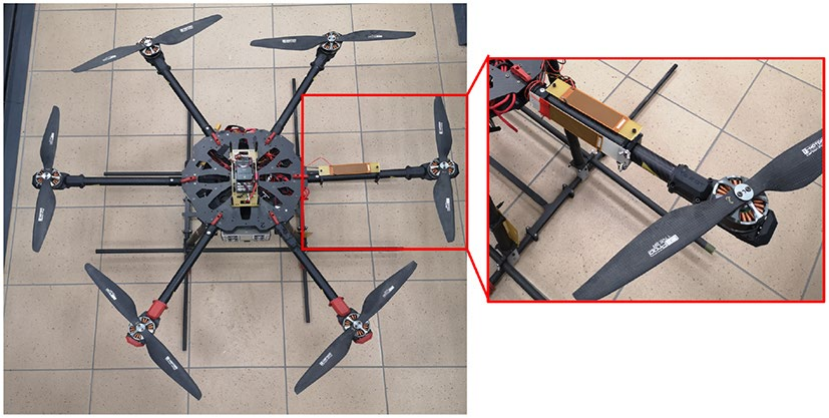
VTOL UAV with both piezoelectric sensors situated in two perpendicular planes of the arm.

The multicopter frame used in the studies is in hexacopter configuration. Investigations have been established in a single arm (see Fig. 1) where the data were collected using the PXI system acquisition data with the control panel. The mentioned BLDC motors can work with different PWM signal duty cycles to reach an adequate rotation speed.

The carbon fiber multicopter arm containing the MN40149 3-phase brushless motor and a propeller of the size of 16 $\times$ 5.4 is chosen for further investigation. Taking this structure into account, the UAV arm was filled by two macro fiber composites of type MFC 8528P2, one of which is located in the upper plane of this arm (*X*–*Z*), while the second is located in the side plane of this arm (*Y*–*Z*). Additionally, the laboratory stand was retrofitted in the system acquisition data PXI, a laser displacement sensor LQ10A65PUQ to measure the vibration of this arm.

### Simulation and main element description

**Figure 2:**
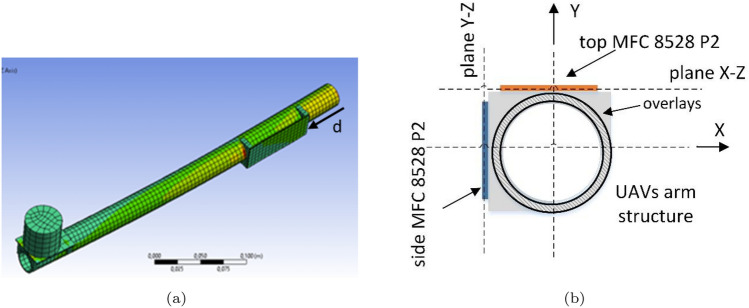
UAVs arm structure with overlay and the piezosensor (**a**) numerical model of, (**b**) cross section.

To define the main location of the piezoelectric sensors in the UAV arm for fault diagnosis, the computational model of this mechanical structure is required. A FEM model of the UAV arm structure with tip mass representing the intact and damaged BLDC motor (Fig. 2a) is investigated to define the initial location of the piezoelectric sensors. This simulation was numerically solved by Harmonic Response Toolbox in Ansys software. The piezoelectric sensors are then located within a distance *d* from the fixed end of the beam/arm, as illustrated in Fig. 2a.

The UAV arm has a circular shape where the piezosensors are with a square shape as illustrated in Fig. 2b. The piezoelectric elements have been attached in the arm using overlays made from carbon fiber materials. The FEM simulation has been elaborated by taking parameters of this structure that are shown in the Table 1 including the material and the dimension of the structures.

**Table 1:** Simulated parameters of the UAVs arm structure with the propulsion system^29^.

Parameter	Tip mass (BLDC motor)		Host structure with overlay	
Material	Aluminum		Carbon fiber	
Radius (m)	${R}_{tip}$	0.0235	${R}_{out}$	0.0252
			${R}_{in}$	0.0219
Length (m)	–		${L}_{a}$	0.390
Thickness (m)	${t}_{tip}$	0.025		
Density (kg/m^3^)	${\rho }_{tip}$	1810	${\rho }_{a}$	1810
Young’s modulus (GPa)	${E}_{tip}$	69	$E_{a}$	75
Poisson ratio	–	0.33	–	0.3

The selected piezoelectric transducer MFC 8528 P2 is based on homogenized Macro Fiber Composite (MFC) with an overall dimension of 103 $\times$ 31 mm, where the active area is approximately 85 $\times$ 28 mm. The piezoelectric transducer has a thickness of 0.003 mm and a density of 4500 kg/m^3^. Additionally, the piezoelement properties are summarized in the Table 2.

**Table 2:** Properties of the piezo sensors type MFC8528 P2^30^.

Young’s modulus (GPa)		Poisson’s ratio (−)		Shear modulus (GPa)		Piezoelectric charge coefficient (pC/N)		Relative Permittivity (−)	
E_x_	31.6	v_xy_	0.4	G_xy_	4.9	d_31_	173	$\epsilon_{r}^{T}$	2253
E_y_	17.1	v_yz_	0.2	G_yz_	2.5	d_32_	150		
E_z_	9.5	v_xz_	0.4	G_xz_	2.4	d_33_	325		

### Analysis of the BLDC motor failure

**Figure 3:**
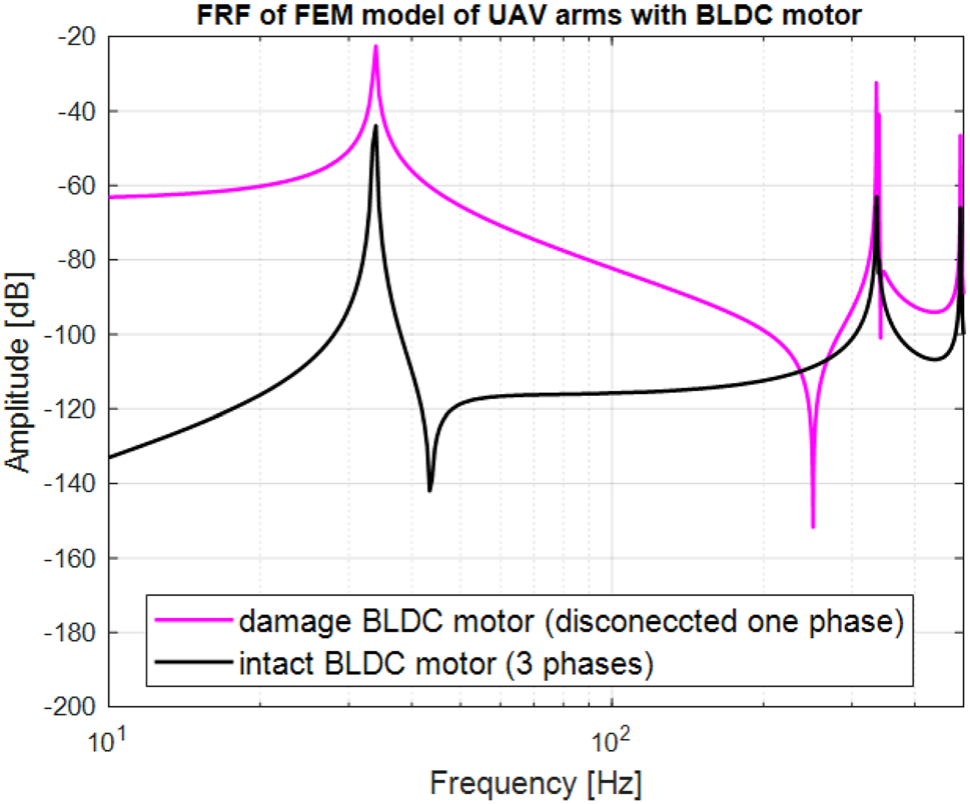
Comparison of the frequency response function of the FEM model of UAV arm with the tip mass representing BLDC motor MN4014-9 excited to vibration by mechanical force corresponding to duty cycle of 40% PWM signal.

The fault on the UAV arms can cause the damage of the BLDC motor by disconnection of at least one of his phases. The frequency response of the FEM model for both intact and damaged propulsion systems have been determined and shown in Fig. 3. It can be seen that frequency vibrations of UAVs arm structure with the intact propulsion system should not exceed 103 Hz where the first resonance of this structure is generated. This upper value of frequency range is related to the BLDC motor voltage supply (set to 15 V) and Kv rating of motor (set to 400 rpm/V). Another behavior can be observed for the damage propulsion system with one disconnected phase. Then, the amplitudes of the first two resonance peaks of UAV arm structure are significantly increasing (difference close to 30 dB), as well as values of the first anti-resonance frequency is shifted to higher frequency. Such a behavior of considering system from structural health monitoring (SHM) point of view can lead to instability of the whole UAV during flight and consequently to appearance of an additional vibration in higher frequencies range. Finally, taking into account all symptoms related to the behavior of the FEM model of the UAV arm, it can be noticed that monitoring of Unmanned Aerial System (UAS) with the intact and damaged brushless motor should be performed in a wider frequency range over 300 Hz.

## Piezoelectric sensor location on the UAV arm

**Figure 4:**
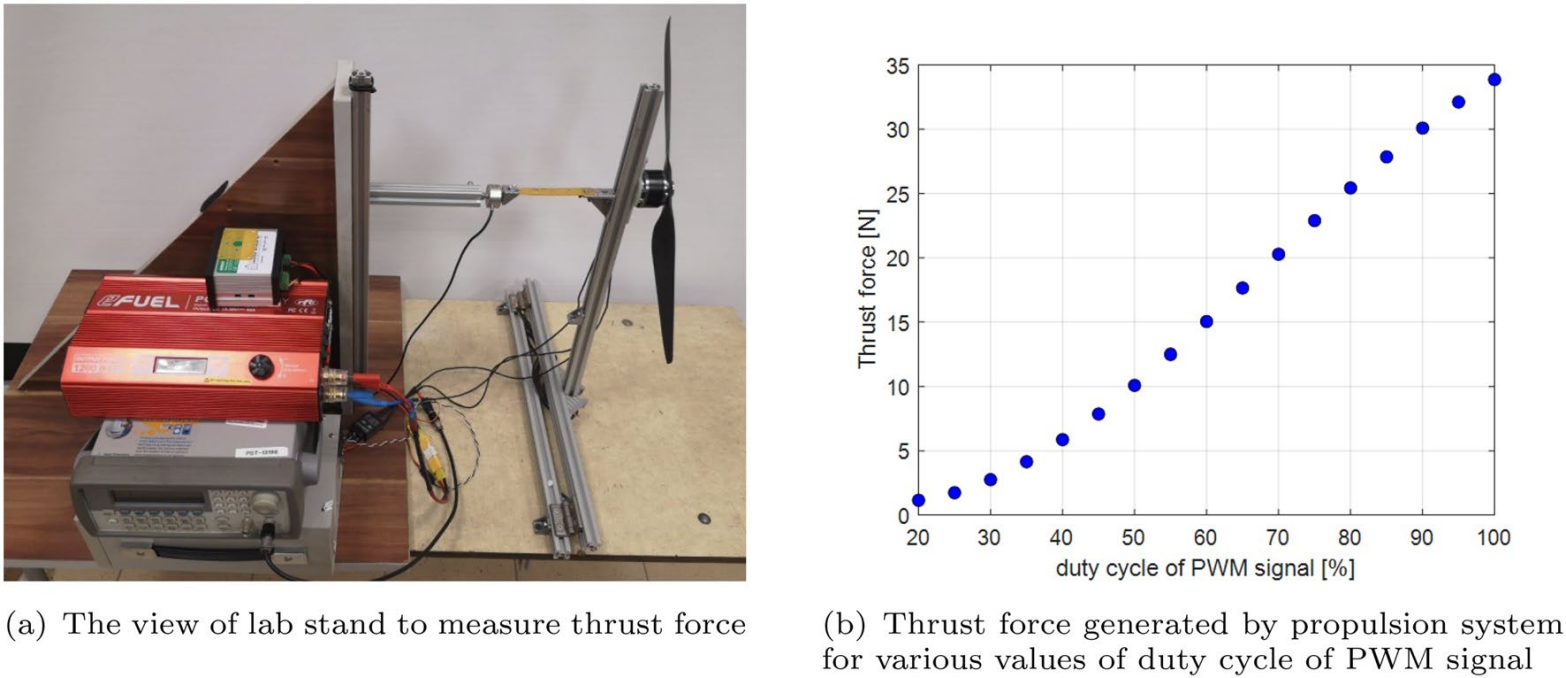
Thrust force of analysed propulsion system.

The location of piezosensors on the UAV arm structure plays a big role in the faster and accurate detection of a fault in the UA system itself. To locate the piezoelements, numerical calculations were performed using the Ansys Workbench transient toolbox, as shown in Fig. 2. This was the first stage of the research. Simulations were established for both FE models of the UAV arm with piezosensor located in two different planes of the *X*–*Z* (top) and *Y*–*Z* (side) for intact and damaged propulsion systems, respectively. Furthermore, the analysis considers the mechanical force with different amplitudes corresponding to the thrust force generated by the propulsion system for each time that is applied to the tip mass representing a real brushless DC motor. Taking this fact into account, numerical calculations of both FEM models are performed for six different amplitudes of thrust force changing in the range of 2.94–12.5 N, which affect the stability of the system during flight. The particular values of this force that correspond to changing the duty cycle of the PWM signal in the range 30–55% are determined on the laboratory stand shown in Fig. 4a. In this case, the PWM signal is generated from the Agilent signal generator and then applied to the UAS located on the L-shape beam by the high current amplifier. Then, the obtained amplitude of thrust force for each value of the PWM signal is measured using a strain sensor and next recorded by the system acquisition data connected to the computer.

**Figure 5:**
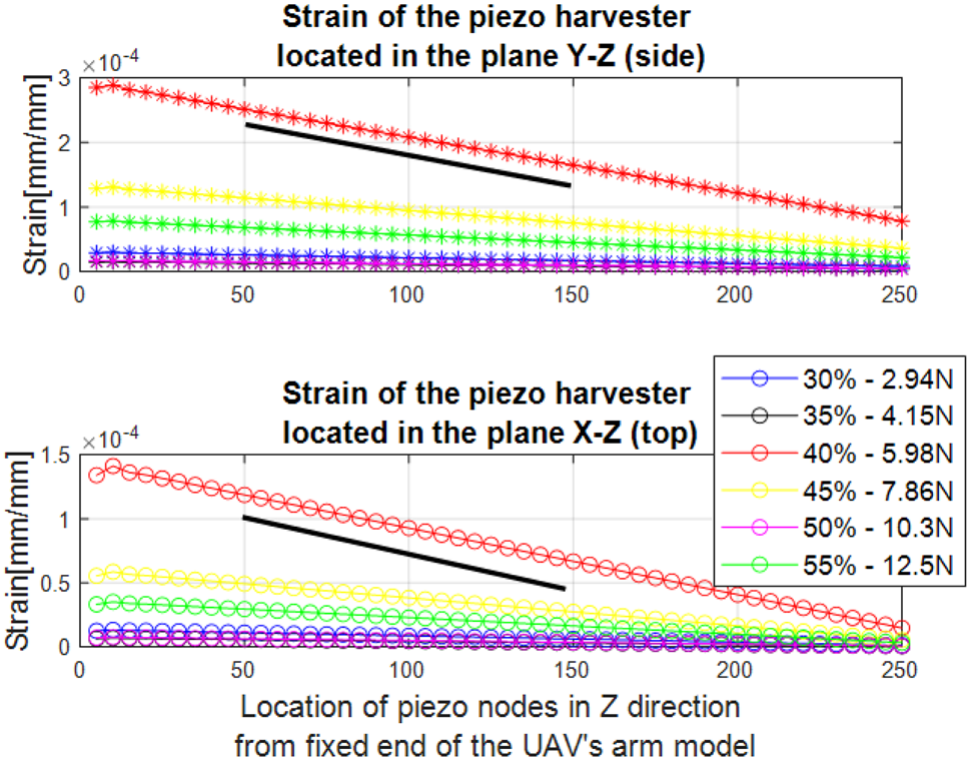
Piezoelectric element strains located on arm of UAV (**a**) in the plane *Y*–*Z*, (**b**) in the plane *X*–*Z*. Thrust force generated by propulsion system for various values of duty cycle of PWM signal for piezo in 50 mm.

In the next stage, the values obtained for the thrust force generated by the propulsion system allowed us to perform an analysis of the mechanical strains for both FE models with piezosensor located in two perpendicular planes *X*–*Z* and *Y*–*Z*, respectively. Observing the results presented in Fig. 5 it can be noticed that the piezoelement located at a distance of 50 mm, considering the size of the UAV platform housing, from the fixed end is more promising for faster detection of the propulsion system fault. Then, the strains of the piezosensor in two considered planes *X*–*Z* and *Y*–*Z* are the highest that reach 0.15 $\upmu$m/mm and 0.3 $\upmu$m/mm, respectively. Another effect can be observed when the piezoelements are located far away from the fixed end (at 250 mm). Then, the strains of the piezosensors in the two planes are significantly reduced. In summary, due to the variation in the PWM duty cycle, the strain changes. It is maximal at 40% PWM, where the force is approximately 5.98 N.

Further analysis of mechanical strains presented in Fig. 5 indicated an increasing amplitude strains of the UAV arm structure with the growth of amplitude of thrust force only up to 6N. This effect is due to increasing the frequency vibration of the mechanical structure to a frequency close to the first resonance frequency.

The measured thrust force from this lab test is shown in Fig. 4b where it goes from 1 N in a duty cycle of 20% to 35 N in a duty cycle 100%. Experiments have been established with a variable PWM duty cycle in the range of 10–100% with step 10%. Further increasing the duty cycle of the PWM signal to 55% leads to generating a higher amplitude of thrust force (12.5 N) and simultaneously decreasing the amplitude of strain for two different locations of the piezosensors in the structure. The reduction in the PWM signal is due to the safety of the researcher and the adjustment of the duty cycle values of the PWM signal to the value needed to maintain UAV stability during flight. The measured thrust force and deformation from both locations of the piezosensors indicated that they are higher when the system that supplies the PWM signal with the duty cycle is greater than 40%, resulting in an inverse effect.

The optimal piezosensor position requires to consider the system dimension including the arm length and the piezoelement length, as well as the strain force that helps for faster detection of fault of the propulsion system damage. On looking at the system dimension, the range of piezoelectric elements can be limited up to 150 mm from the fixed end of the arm. On the other hand, the sensitivity for fast detection can be guaranteed when the strain is greater than 0.1 $\upmu$m/mm. Based on these considerations, as well as the results shown in Fig. 5, the optimal piezosensors in the real structure were selected at a distance of 50 mm from the fixed end of this arm. In this position, the strain of the top piezoelement is about 0.01 $\upmu$m/mm, 0.12 $\upmu$m/mm, 0.007 $\upmu$m/mm for PWM duty cycle of 30%, 40% and 50%, respectively. Additionally, the strain of the side piezoelement is about 0.02 $\upmu$m/mm, 0.25 $\upmu$m/mm, 0.01 $\upmu$m/mm for PWM duty cycles of 30%, 40% and 50%, respectively. It can be noticed that the amplitude strains of piezosensors located in the side plane of the UAV arm (*Y*–*Z*) are almost twice as high as those located in the perpendicular plane (*X*–*Z*).

## Analysis on the amount of piezoelectric sensors

### Piezosensor output voltage in the time domain

**Figure 6:**
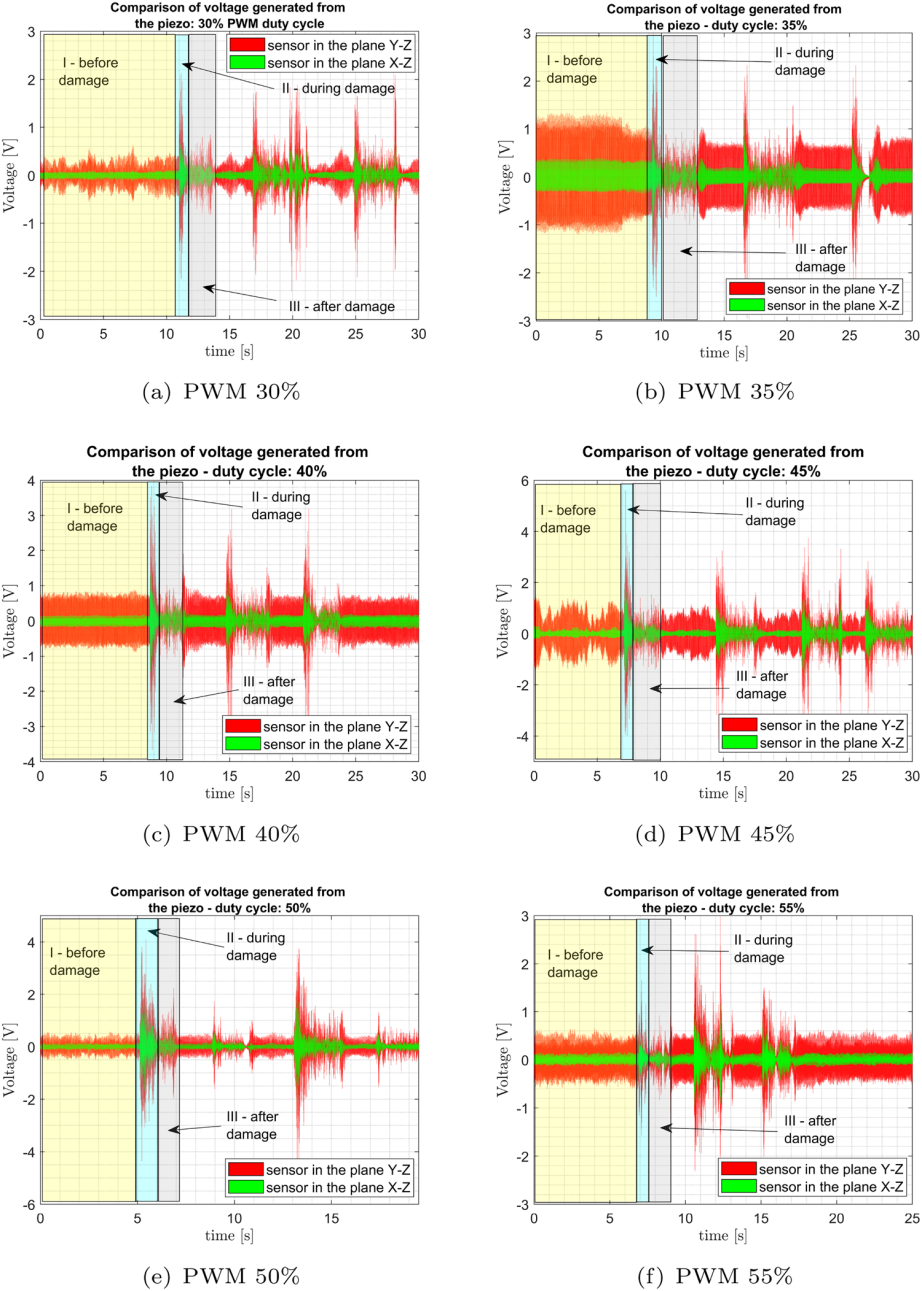
Piezosensors output for different duty cycles (a)30%, (**b**) 35%, (**c**) 40%, (**d**) 45%, (**e**) 50%, (**f**) 55%.

The presented effect of damage on the motor is also related to the duty cycles of PWM signals as shown in Fig. 1 where the piezosensors are located at a distance of 50 mm from the frame base of the VTOL UAV. It can be seen that the amplitude of voltage generated by both piezosensors before and after the damage for the duty cycle between 30 and 55% with step 5% is illustrated in Fig. 6a–f, respectively. The highest voltage amplitudes are obtained for the UAS that supplies the PWM signal with duty cycle equal to 40%, and further increasing the duty cycle of PWM over 40% leads to an inverse effect and decreases the voltage amplitude generated by both piezosensors. By comparing the response of both piezoelectric sensors in Fig. 6, the piezosensor located in the *Y*–*Z* plane is more sensitive to the other piezosensor placed in the *X*–*Z* plane. In fact, the piezosensor located in the *Y*–*Z* plane generated a voltage of up to 0.5 V under normal conditions and increases the voltage about 1.2 V time for 30%, to 45%, respectively. However, the piezosensor located in the *X*–*Z* plane increases the voltage to 0.1 V under normal conditions and increases the voltage to about 0.1 V and 0.2 V time for 30%, to 55%, respectively. Furthermore, it can be noticed that the damage to the propulsion system verifies the ability of the whole structure, and this effect can be more visible in the plane *Y*–*Z* where the amplitude of chaotic vibrations of the UAV arm structure is higher. It properly verifies numerical results and indicates generation of periodic vibrations. Further analysis of the diagrams shown in Fig. 6 allows us to observe that the fault propulsion system is detected faster by the piezosensor located in the *Y*–*Z* plane. It is shown especially in these regions of time series plots where the voltage peaks generated by this sensor significantly increase, and the amplitudes of these peaks are significantly higher than those measured by the piezosensor located in the *X*–*Z* perpendicular plane. It allows us to notice that injecting the damage to the propulsion system again can lead to instability of the whole structure, and this effect can be more visible in the plane *Y*–*Z* where the amplitude of chaotic vibrations of the UAV arm structure is higher.

### Piezosensor output voltage in the frequency domain

**Figure 7:**
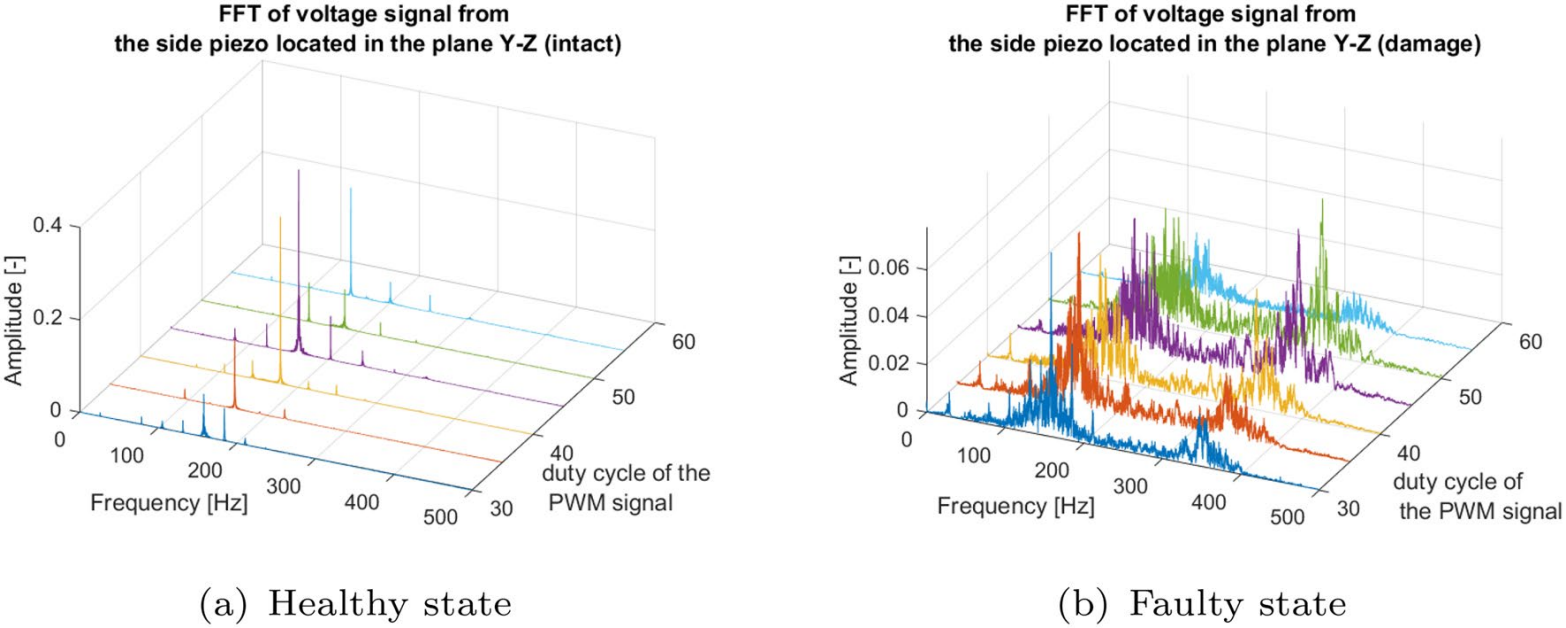
Frequency spectra of voltage signals for the (**a**) intact and (**b**) damage propulsion system measured by the piezosensor located in the plane *Y*–*Z*.

**Figure 8:**
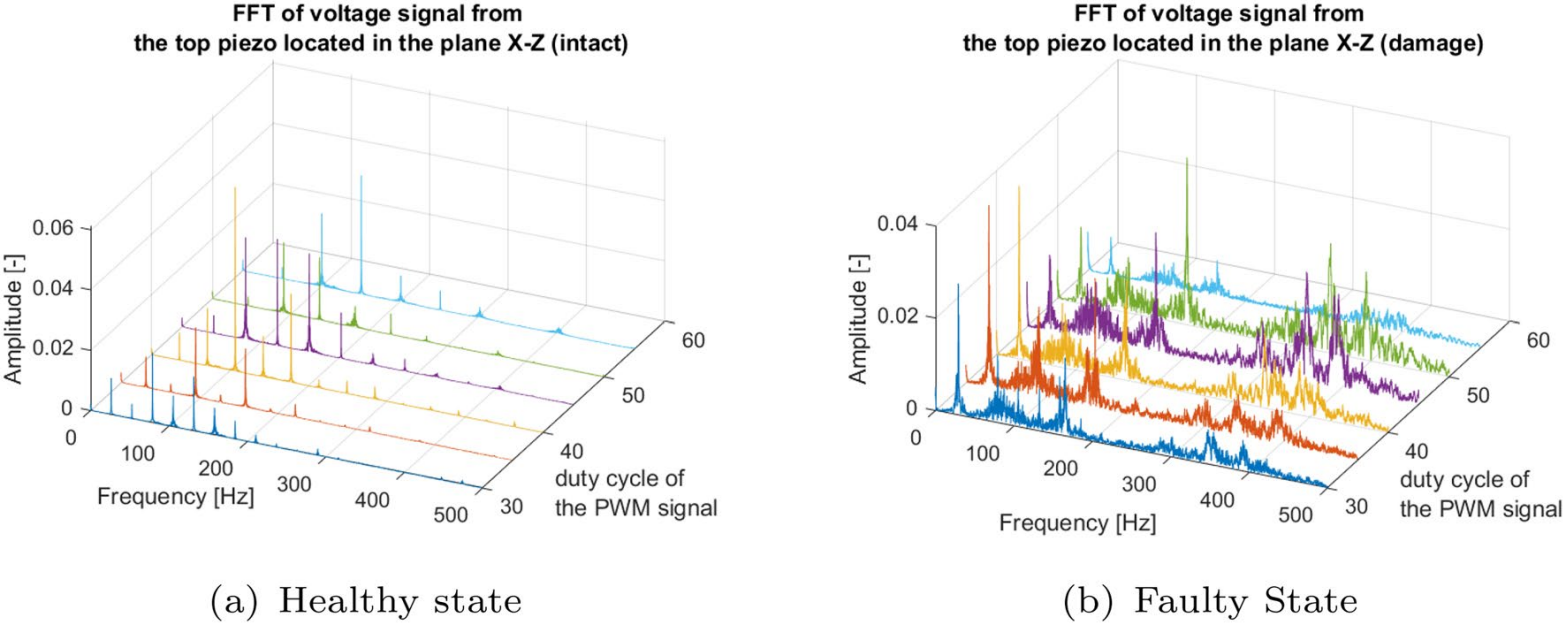
Frequency spectra of voltage signals for the (**a**) intact and (**b**) damage propulsion system measured by the piezo located in the plane *X*–*Z*.

In this section, the behavior of the piezosensors located on the UAV arm in two perpendicular planes for the intact and damaged UAV propulsion system is also analyzed in the frequency domain. To do this, a Fourier analysis was performed for both types of piezosensor locations by varying the values of the change in the PWM duty cycle in the range 30–55%. The results obtained for the piezosensor located in the plane *Y*–*Z* are presented in Figs. 7a and 8a for the piezosensor located in the perpendicular plane *X*–*Z*. In the signals from the damaged motor, a high level of noise appears in the frequency spectra (Figs. 7b, 8b). Furthermore, while damage occurs, a relatively high amplitude in the frequencies between 300 and 450 Hz appears compared to the intact population. These results show that the frequency domain analysis has a great impact on the identification of failure with piezoelectric elements situated in the *Y*–*Z* or *X*–*Z* planes.

The results of Figs. 7 and 8 have been summarized in detail in the Tables 3 and 4 for the piezoelectric elements in the *Y*–*Z* and *X*–*Z* planes, respectively. Analysis allows to indicate significantly higher amplitudes of peaks generated by the side piezosensor (plane *Y*–*Z*) in comparison to those generated by the top piezosensor (plane *X*–*Z*) for all values of duty cycle of the PWM signal applied to intact and damaged propulsion system, respectively. The maximum values of peak voltage for both piezosensors for the intact and damaged systems do not change much. Furthermore, it is observed that the failure of the UAV propulsion system leads to generating mainly amplitude peaks in the frequency range between 70 and 180 Hz with intact and defected systems. This makes the analysis of the amplitude peak and their frequencies not as expressive parameters to consider for fault identification.

**Table 3:** Summary of the frequency response with the piezo sensor located in the *Y*–*Z* plane.

PWM duty cycle	Freq. excit. (Hz)	Output from the intact system			Output from the damage system		
		Amp. (−)	Freq. (Hz)	Freq. range (Hz)	Amp. (−)	Freq.(Hz)	Freq. range (Hz)
30%	26.30	0.093	158.19 (6 $\times$ f_exc_)	200	0.051	158.90	400
35%	31.77	0.151	158.76 (5 $\times$ f_exc_)	210	0.056	156.90	400
40%	35.63	0.398	178.18 (5 $\times$ f_exc_)	200	0.077	143.65	400
45%	40.63	0.362	162.53 (4 $\times$ f_exc_)	200	0.073	148.90	400
50%	45.53	0.077	136.60 (4 $\times$ f_exc_)	220	0.044	149.80	400
55%	50.34	0.234	151.12 (3 $\times$ f_exc_)	220	0.026	149.80	400

**Table 4:** Summary of the frequency response with the piezo sensor located in the *X*–*Z* plane.

PWM duty cycle (%)	Freq. excit. (Hz)	Output from the intact system			Output from the damage system		
		Amp. (−)	Freq. (Hz)	Freq. range (Hz)	Amp. (−)	Freq.(Hz)	Freq. range (Hz)
30	26.30	0.023	78.90 (3 $\times$ f_exc_)	200	0.028	29.14	400
35	31.77	0.022	95.31 (3 $\times$ f_exc_)	210	0.037	29.14	400
40	35.63	0.061	106.31 (3 $\times$ f_exc_)	200	0.039	29.14	400
45	40.63	0.035	121.00 (3 $\times$ f_exc_)	200	0.016	164.53	400
50	45.53	0.020	136.70 (3 $\times$ f_exc_)	220	0.016	164.53	400
55	50.34	0.039	151.00 (3 $\times$ f_exc_)	220	0.008	29.14	400

### Nonlinear time series investigations

Nonlinear methods like Recurrence Plots (*RP*), Recurrence Quantification Analysis of recurrence plots (*RQA*), and Principal Component Analysis (*PCA*) are investigated to show the sensitivity of fault detection. *RP* is the technique for visualizing the recurrence of states in *m*-dimensional phase space and it helps analyzing the behavior of the dynamic system^27^. The repeatability of a state at time * i* at a different time *j* is marking with black dots in the recurrence plot, while the vertical and horizontal axes represent time. As a result, the recurrence plot (*RP*) can be described in Eq. (1) where *N* is the number of states under consideration, $\varepsilon$ is a threshold distance, $\left\| ..\right\|$ is a norm, ($\cdot$) is the Heaviside function.

1 $$\begin{aligned} \begin{array}{cc} {R_{i,j} =\theta \left( \varepsilon -\left\| x_{i} -x_{j} \right\| \right) ,}&{x_{i} \in \textrm{R}^{m} ,i,j \ldots N} \end{array} \end{aligned}$$

In order to determine the recurrence plots, the 3D attractor is needed and its value should be assessed based on the time delay ($\tau$) estimation. The method used to determine $\tau$ is the mutual information method^33,34^. In this method, the first minimum of the following function is treated as the proper value of $\tau$:

2 $$\begin{aligned} I\left( x(t),x(t+\tau )\right) =\sum _{i,j}p_{i,j} (\tau )\log \left( \frac{p_{i,j} (\tau )}{p_{i} p_{j} } \right) \end{aligned}$$

where $$I(x\left( t\right) ,x(t+\tau ))\ $$is the mutual information function between the original signal and the delayed time series, $p_i,\ p_j$ is the probability that *x*(*t*) is in bin $$i,\ j\ $$of the histogram constructed from the data points in $x,\ p_{i,j}(\tau )$ is the probability that *x*(*t*) is in bin *i* and $x(t+\tau )$ is in bin *j*.

To obtain the attractor reconstruction, the proper embedding dimension (*m*) is calculated using the false nearest neighbors (*FNN*) algorithm^35^. The number of adjacent points in the embedding space changes with the increase in embedding dimension. Disappearing points with the embedding dimension increase are called false neighbors. The dimension for which the fraction of false neighbors is zero is treated as the proper embedding dimension.

**Figure 9:**
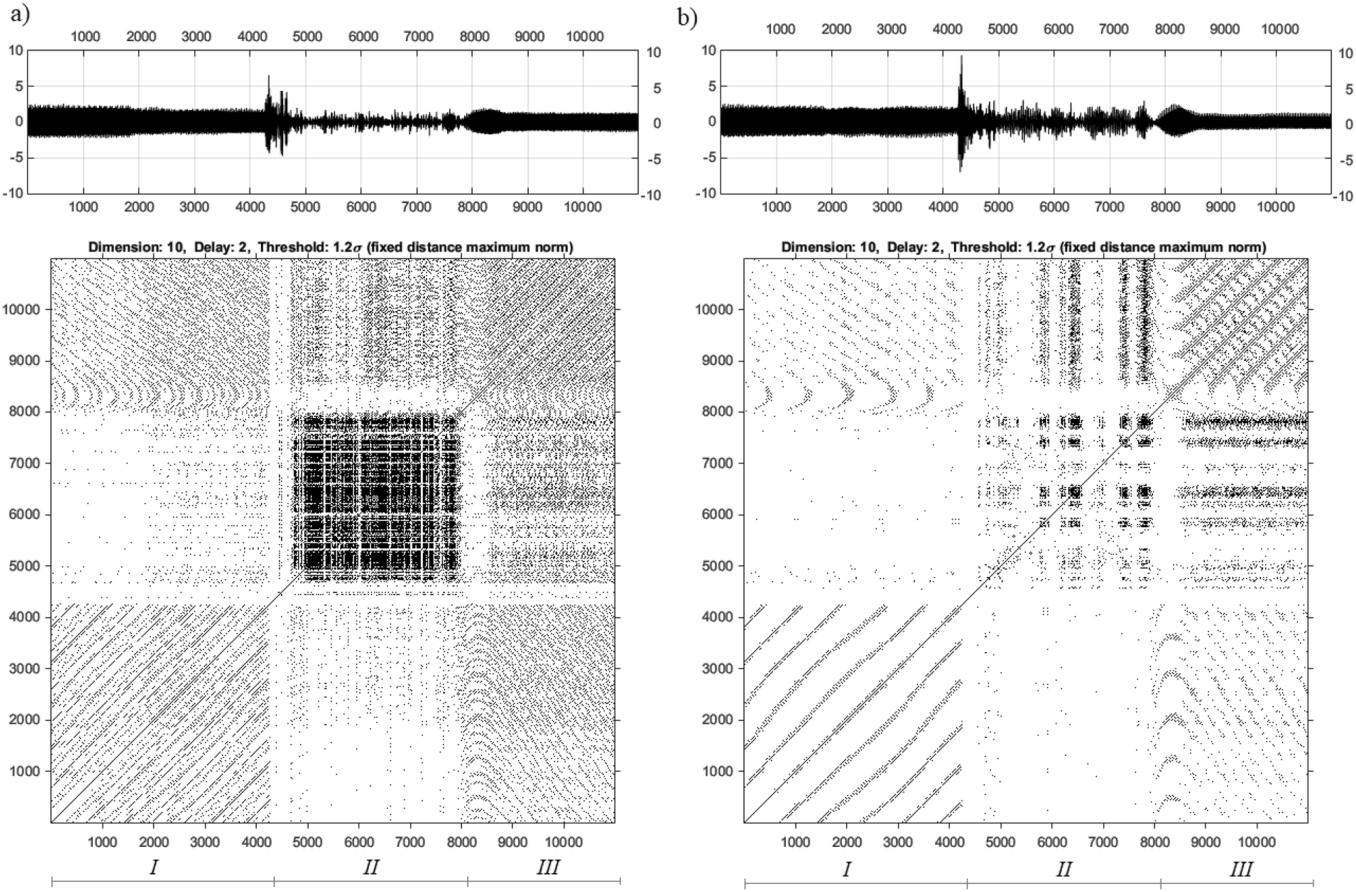
Recurrence plots determined for corresponding voltage signals for piezo harvester located in the side (**a**) and top (**b**) side with duty cycle 35%.

**Figure 10:**
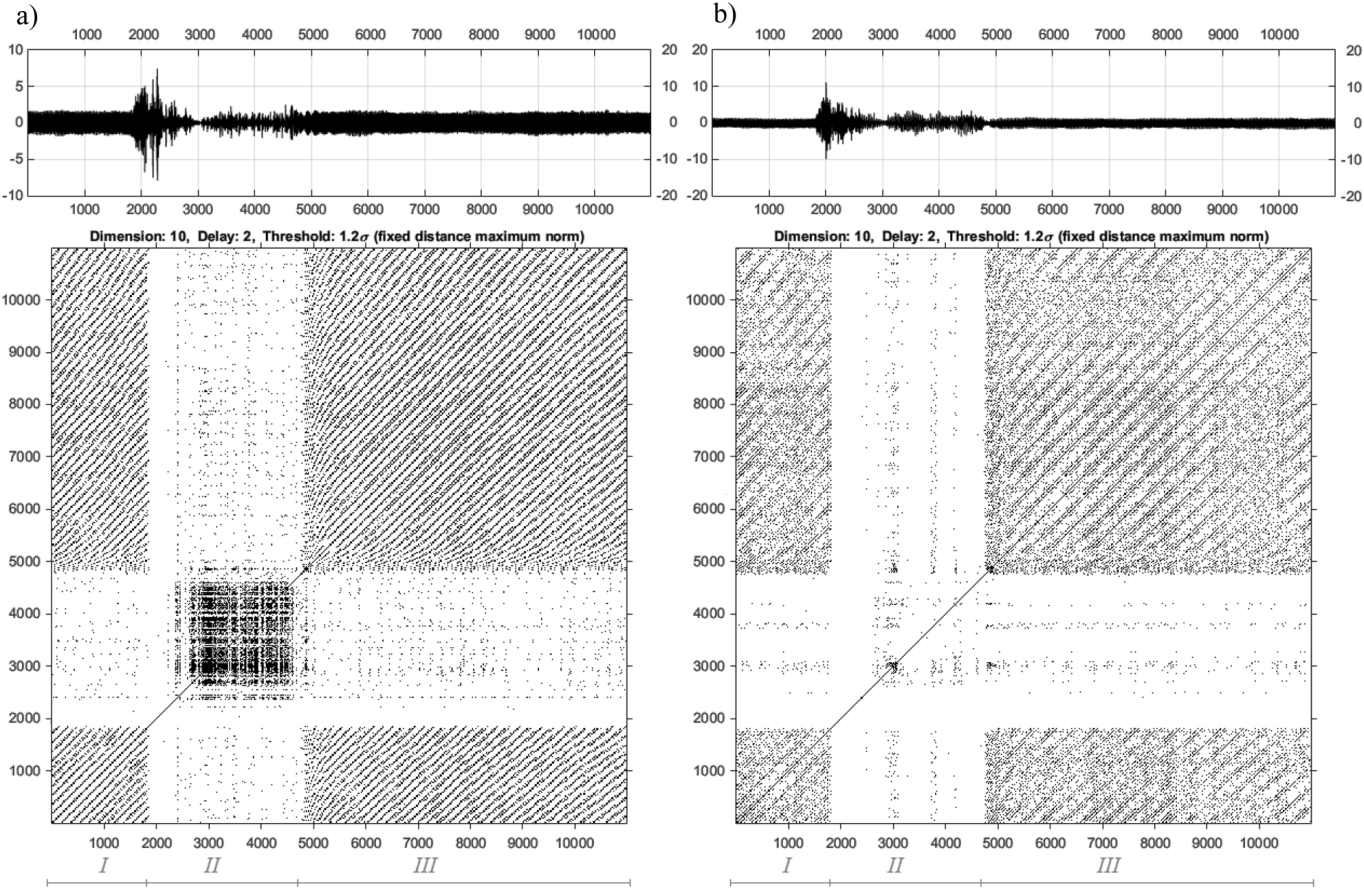
Recurrence plots determined for corresponding voltage signals for piezo harvester located in the side (**a**) and top (**b**) side with duty cycle 40%.

**Figure 11:**
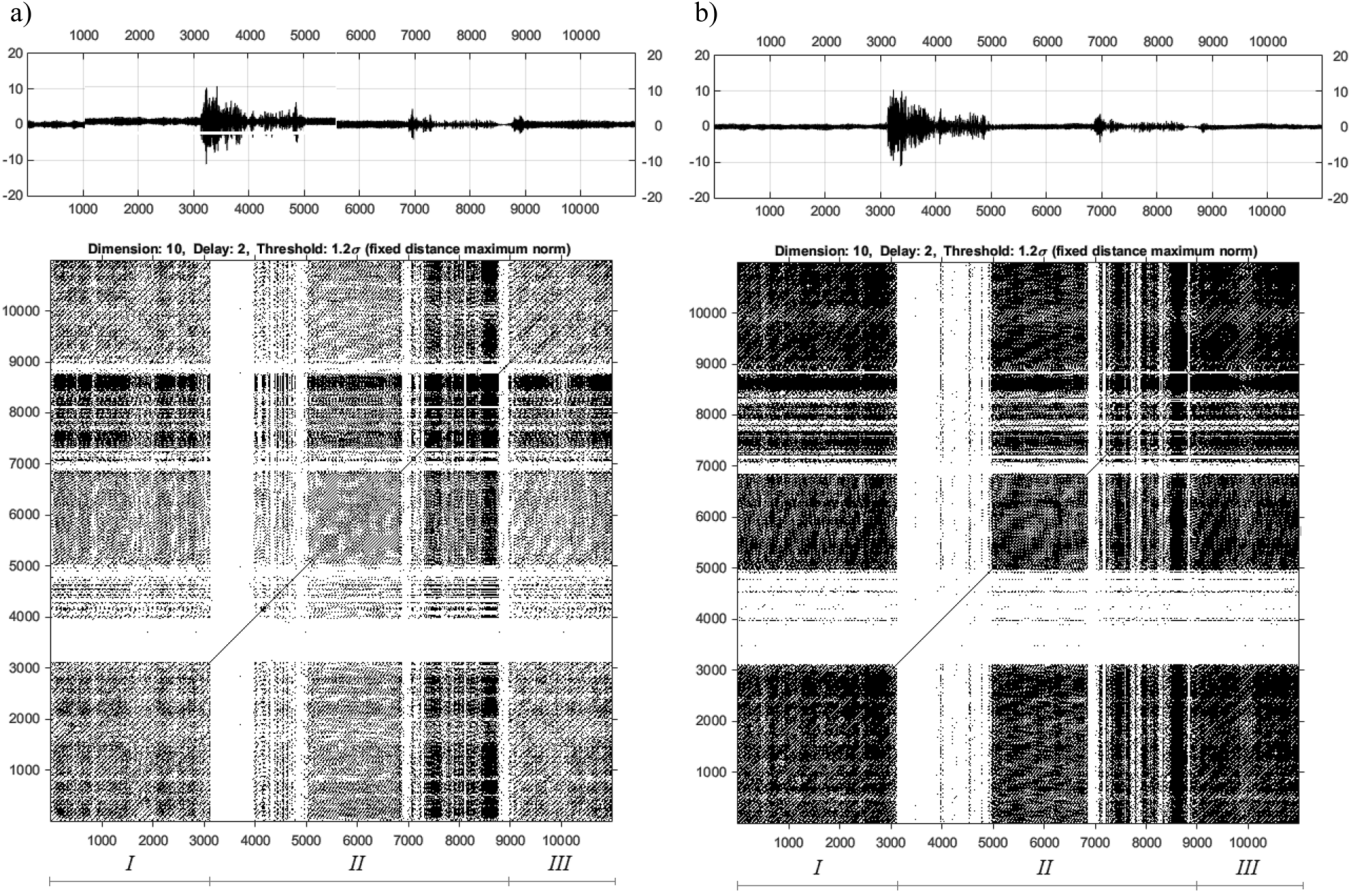
Recurrence plots determined for corresponding voltage signals for piezo harvester located in the side (**a**) and top (**b**) side with duty cycle 50%.

The number of recurrence points in the RP is also related to the threshold value ($\varepsilon$)^35^. The methods of estimation of the $\varepsilon$ value have been considered by the authors in articles^36–39^. In the case discussed above, the analysis of the UAV arm structure with intact and damage to the propulsion system was carried out using nonlinear methods for three chosen values of the PWM duty cycle 35%, 40%, 50%. Taking into account the estimated values of *m*, $\tau$ and $\varepsilon$ collected in Table 5 as well as voltage signals recorded by both piezosensors (see Fig. 6), recurrence plots are determined and shown in Figs. 9, 10, and 11.

**Table 5:** Assumed values of *m*, $\tau$ and $\epsilon$ parameters regarding recurrence analysis method.

PWM duty cycle (%)	$m (-)$	$\tau (-)$	$\epsilon (-)$
35	10	2	1.2
40	10	2	1.2
50	10	2	1.2

Presented in Figs. 9, 10, and 11 RPs were made in the cross-recurrence plot toolbox proposed in^30^. Observing these diagrams, it can be seen that all represent recurrence plots of the UAV arm with the intact and fault UA system supplied by the PWM signal with three chosen values of the PWM duty cycles 35% 40%, 50% respectively. To ease their analysis diagrams of recurrence plots generated on the basis of voltage signals recorded by the side piezosensor (plane *Y*–*Z*) are located on the left side in Figs. 9, 10, and 11, but those obtained with the use of the piezosensor located on the top of the UAV arm (plane *X*–*Z*)—on the right side of Fig. 9, 10, and 11. Furthermore, all diagrams are divided into three sections marked by *I, II, III* where each of them corresponds to the intact propulsion system, the damage system, and again the intact system.

The analysis of recurrence plots provides a qualitative assessment of the nature of the dynamic response of the system. Long diagonal lines indicate the regular nature of vibrations, while short diagonal lines or isolated points indicate chaotic vibrations. Analysis of Section *I* (intact propulsion system) allows us to indicate that the measured voltage time series in two perpendicular directions of the UAV arm are periodic. In effect, a high repeatability of recorded voltage signals leads to generating a lot of black points on the diagrams. Different behavior is obtained in Section * II* where the fault of the propulsion system leads to the generation of chaotic vibrations in two perpendicular planes *X*–*Z* and *Y*–*Z* and consequently to the disappearance of the black points on the diagrams. Considering this fact, it can be observed that the increase in duty cycle of the PWM signal from 35 to 50% leads to the appearance of alternating chaotic and periodic vibrations of the structure. This behavior of the structure is shown especially in Fig. 11b for 50% PWM where the group of black dots on the diagram is separated by white vertical lines. Still others’ behavior can be observed for the structure with a propulsion system supplied by the PWM signal with 40% duty cycle. Then damage to the BLDC motor located at the free end of the UAVs arm leads to total disappearance of periodic vibrations mainly especially in the side plane of the UAVs arm and consequently to generate a recurrence plot in Section *II* without black dots. Summarizing these diagrams, it can be concluded that the appearance of a wide white band on the recurrence plot without black dots can be recognized as a potential indicator of a fault of the propulsion system.

Next, the recurrence quantification analysis method (RQA) is used to perform a deeper analysis of the recurrence plots, as well as to determine the correct location of the piezosensor in the UAV arm structure, allowing faster fault detection of the propulsion system^31^. Taking this method into account, two recurrence quantification recurrence rates (RR) given by Eq. (3) and determinism (DET) given by Eq. (4) are determined^31^.

3 $$\begin{aligned} RR & = \frac{1}{N^2}\sum ^N_{i,j=1}{R^{\varepsilon }_{i,j}} \end{aligned}$$

4 $$\begin{aligned} DET & = \frac{\sum ^N_{l=l_{min}}{lP(l)}}{\sum ^N_{l=1}{lP(l)}} \end{aligned}$$

**Figure 12:**
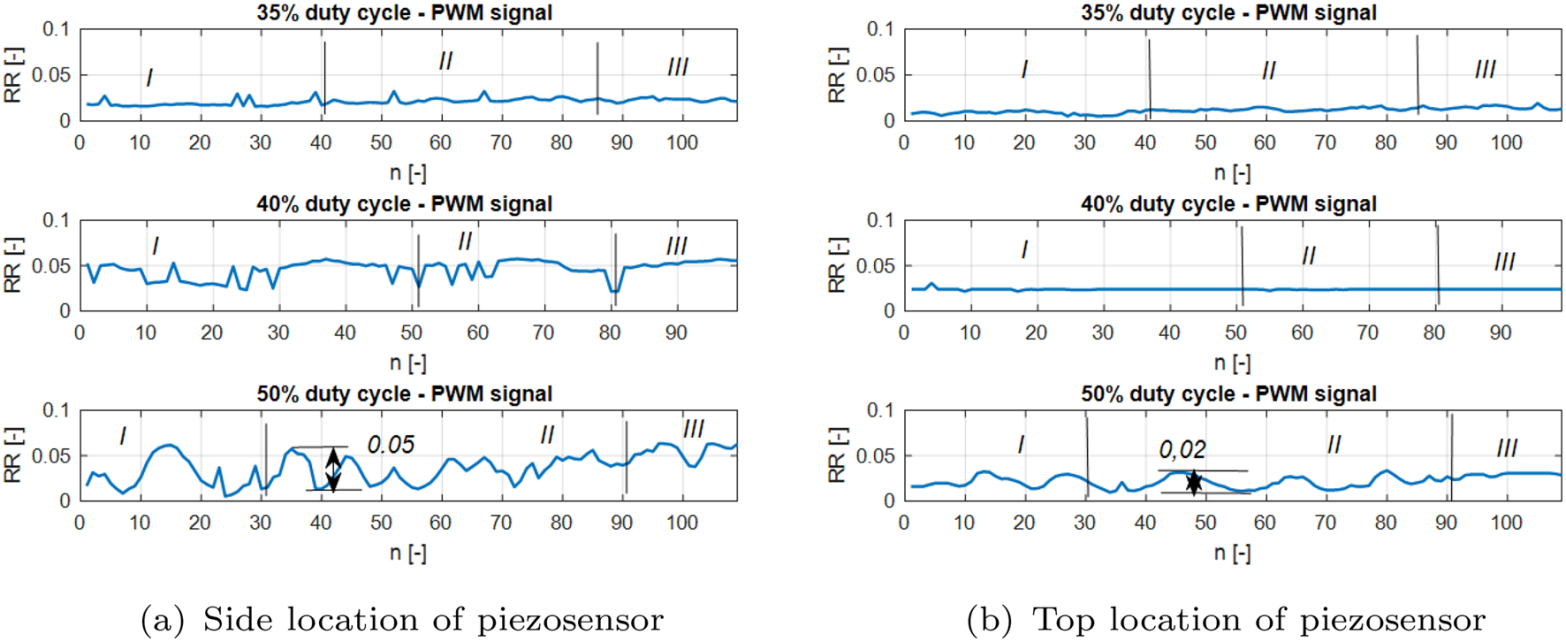
Recurrence quantification RR for chosen PWM duty cycles of 35%, 40% and 50% applied to the UAV system piezosensor located in (**a**) piezosensor located in the plane Y–Z (side), (**b**) piezosensor located in the plane X–Z (top).

**Figure 13:**
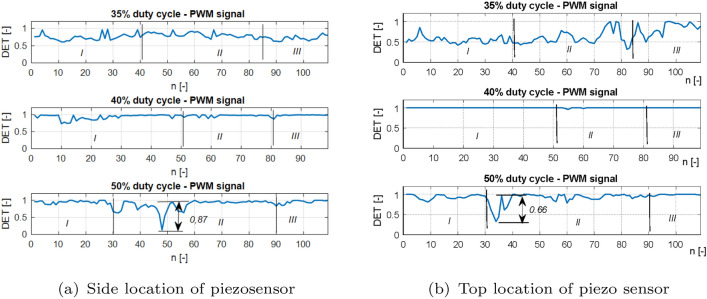
Recurrence quantification DET for chosen PWM duty cycles—35%, 40% and 50% applied to the UAV system (**a**) piezosensor located in the plane Y–Z (side), (**b**) piezosensor located in the plane X–Z (top).

The aforementioned coefficients in the considered case are calculated for subsequent time windows with constant length, where a single window is shifted by a constant number of samples. According to this method, the length of the recorded voltage signals for the aforementioned PWM duty cycle 35%, 40%, 50% were equal to 11,000 samples (11 s) where a single window size was set, 100 samples (0.1 s) were shifted by 10 samples (0.01 s). It leads to obtaining plots of *RR* and *DET* value changes corresponding to the voltage signals in Fig. 6 that are shown in Figs. 12 and 13, respectively.

**Figure 14:**
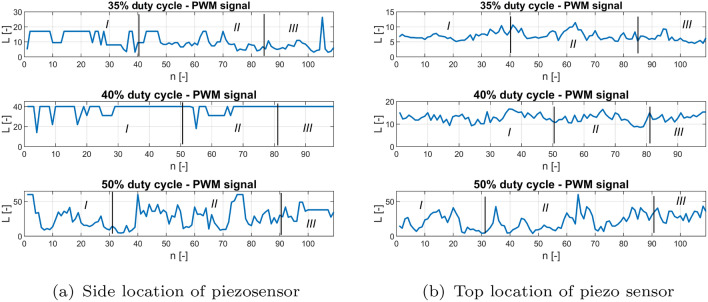
Recurrence quantification L for chosen PWM duty cycles—35%, 40% and 50% applied to the UAV system (**a**) piezosensor located in the plane Y–Z (side), (**b**) piezosensor located in the plane X–Z (top).

**Figure 15:**
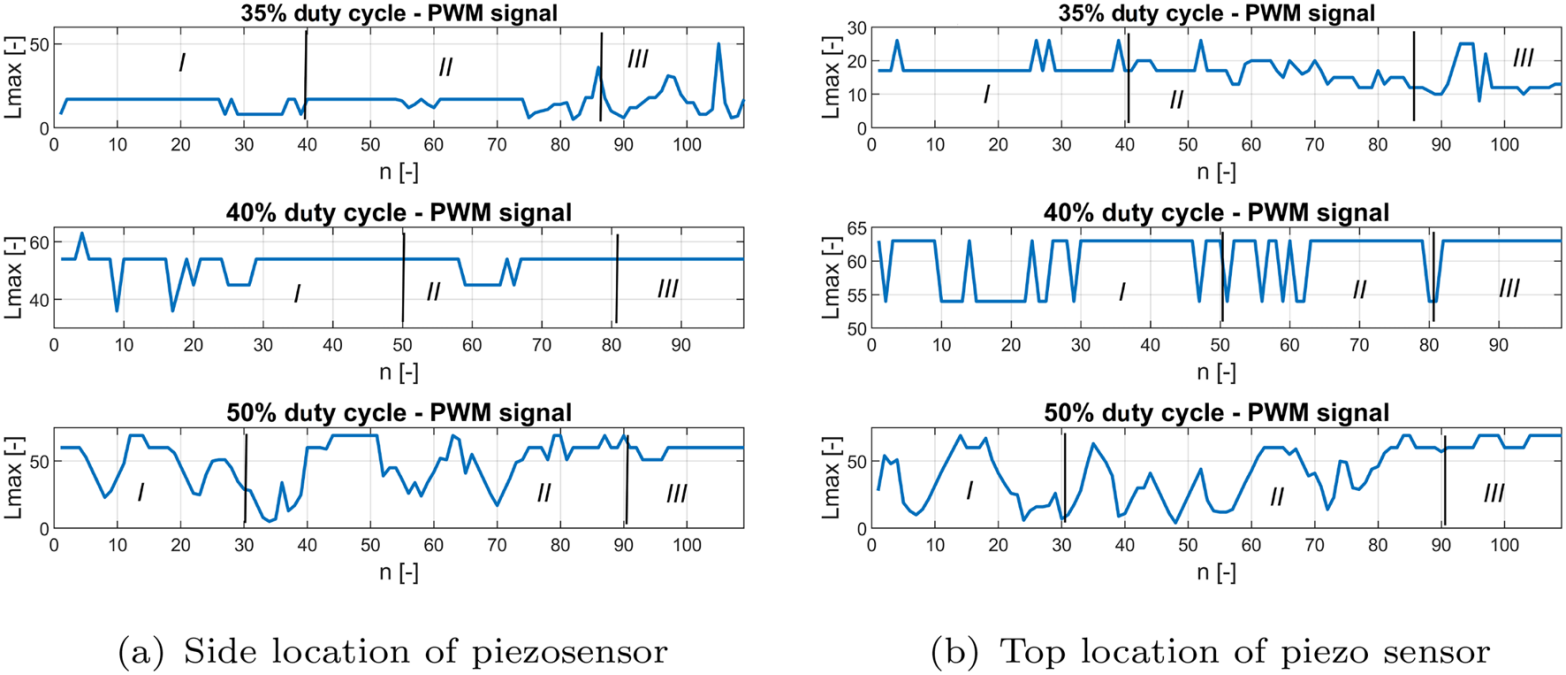
Recurrence quantification ${L}_{max}$ for chosen PWM duty cycles—35%, 40% and 50% applied to the UAV system (**a**) piezosensor located in the plane Y–Z (side), (**b**) piezosensor located in the plane X–Z (top).

Taking into account Figs. 12 and 13 it can be observed that particular diagrams of recurrence quantifications *RR* and *DET* calculating for a constant values *m*, $\tau$ and $\varepsilon$ parameters (see Table 5) are located in two columns where each of them corresponds to the location of the piezosensor on the UAV arm. The recurrence quantifications *RR* and *DET* determined for the recorded voltages from the MFC element located in the plane *Y*–*Z* are collected in the first column of both figures (see Figs. 12a, 13a), while those determined for voltage signals measured with the help of the piezosensor located in the plane *X*–*Z* collecting in the second column. Similarly, as was presented in Figs. 9, 10, and 11 also, in this case, the horizontal axis is divided into three Sections *I, II* and *III* where each of them corresponds to an intact propulsion system, a damaged propulsion system, and again intact UAS after temporary damage, respectively.

Analysis of these diagrams showed that the side location of the piezosensor on the UAV arm is a better placement to harvest energy from vibrations and consequently faster detect the propulsion system fault by chosen value of PWM duty cycle. It is shown especially in Fig. 13a—section *II* for the system with 50% duty cycle where introducing temporary damage to the propulsion system leads to an increase in the amplitude of the vibration structure and generates relatively higher values of DET for recurrence quantification in comparison to the values of DET shown in Fig. 13b—section *II*. Similar effects can be observed during the analysis of * RR* presented in Fig. 12a for 50% PWM where its relative values (see Section *II*) are greater than those shown in Fig. 12b—Section *II*.

Additionally, two other quantificators referred to the diagonal lines of recurrence plots are taken into account, i.e. average length of the diagonal line L and maximal length of the diagonal line Lmax. The results of mentioned quantificators are presented in Figs. 14 and 15, both quantificators refer to the periodicity of the time-series, the longer the diagonal line, the more periodic response is. Somehow, they confirm small value of determinism in Section *II*. The response has a greater tendency to fluctuate in mentioned section. Moreover, it can be observed that this fluctuation is increasing with the level of PWM signal, what refers to stronger impact on the UAV’s structure. In this case, additional two recurrence quantificators will be taken into account in the PCA analysis.

Other behaviors can also be observed for systems supplied by the PWM signal with duty cycle 40%. Then, the relative values of both recurrence quantifications *RR* and *DET* are unchanged and close to the unit for the intact as well as the damage propulsion system. In order to find an explanation for this behavior of this system, further analysis is performed in two steps. In the first, the obtained values of both recurrence quantifications were compared with five other samples of the same recorded voltage time series by assuming the same parameters collected in Table 5. Then, the obtained results for all analyzed voltage time series from both piezo sensors indicate that perturbations of *RR* and *DET* values are still small and further analysis of this system for still other time series should be omitted. In the second step, the threshold value is reduced. It leads to an increase in relative values of *RR* and *DET *that could be result of occurring vibrations of the considered structure with almost constant amplitude. It means that the 40% duty cycle of the PWM signal applied to the propulsion system leads to vibrations of the UAVs arm with a frequency close to the natural frequency and consequently to the appearance of periodic and strongly repeatable voltage signals recorded by both piezosensors.

### Statistical approach by principal component analysis

In this Section, the principal component analysis (PCA) for two different piezosensor locations on the UAV arm structure is used to verify the previous conclusion^35^. In the paper, the PCA was calculated using the built-in Matlab Princomp function^36^. In order to do this, two input matrices ***X***_side_ and **X**_top_ are defined firstly for two different piezo locations on the UAV arm structure where each of them consists of two previous recurrence quantifications RR, DET as well as two other quantificators like the average length of the diagonal length (*L*) and the length of longest diagonal line (*Lmax*). Two last quantificators are expressed in the following forms^35^:

5 $$\begin{aligned} L & = \frac{\sum ^N_{l=l_{min}}{lP(l)}}{\sum ^N_{l=l_{min}}{P(l)}} \end{aligned}$$

6 $$\begin{aligned} L_{max} & = \textrm{max}\mathrm (\{l_i,i=1,\dots ,N_l\}) \end{aligned}$$

where *P(l)*—histogram of diagonal line lengths, *N*—maximal number of diagonals. Next, the covariance matrices **S**_top_ and  **S**_side_ given by Eqs. (7) and (8) allow to perform their decomposition and determine two separate feature matrices with the most valuable components for three chosen PWM duty cycles 35%, 40%, 50% supplied to the propulsion system.

7 $$\begin{aligned} {S}_{top} & = \left[ \begin{array}{cc} \begin{array}{cc} cov(x_{DET\_top},x_{DET\_top}) & cov(x_{DET\_top},x_{RR\_top}) \\ cov(x_{RR\_top},x_{DET\_top}) & cov(x_{RR\_top},x_{RR\_top}) \end{array} & \begin{array}{cc} cov(x_{DET\_top},x_{L\_top}) & cov(x_{DET\_top},x_{L_{max}\_top}) \\ cov(x_{RR\_top},x_{L\_top}) & cov(x_{RR\_top},x_{L_{max}\_top}) \end{array} \\ \begin{array}{cc} cov(x_{L\_top},x_{DET\_top}) & cov(x_{L\_top},x_{RR\_top}) \\ cov(x_{L_{max}\_top},x_{DET\_top}) & cov(x_{L_{max}\_top},x_{RR\_top}) \end{array} & \begin{array}{cc} cov(x_{L\_top},x_{L\_top}) & cov(x_{L\_top},x_{L_{max}\_top}) \\ cov(x_{L_{max}\_top},x_{L\_top}) & cov(x_{L_{max}\_top},x_{L_{max}\_top}) \end{array} \end{array} \right] \end{aligned}$$

8 $$\begin{aligned} {S}_{side} & = \left[ \begin{array}{cc} \begin{array}{cc} cov(x_{DET\_side},x_{DET\_side}) & cov(x_{DET\_side},x_{RR\_side}) \\ cov(x_{RR\_side},x_{DET\_side}) & cov(x_{RR\_side},x_{RR\_side}) \end{array} & \begin{array}{cc} cov(x_{DET\_side},x_{L\_side}) & cov(x_{DET\_side},x_{L_{max}\_side}) \\ cov(x_{RR\_side},x_{L\_side}) & cov(x_{RR\_side},x_{L_{max}\_side}) \end{array} \\ \begin{array}{cc} cov(x_{L\_side},x_{DET\_side}) & cov(x_{L\_side},x_{RR\_side}) \\ cov(x_{L_{max}\_side},x_{DET\_side}) & cov(x_{L_{max}\_side},x_{RR\_side}) \end{array} & \begin{array}{cc} cov(x_{L\_side},x_{L\_side}) & cov(x_{L\_side},x_{L_{max}\_side}) \\ cov(x_{L_{max}\_side},x_{L\_side}) & cov(x_{L_{max}\_side},x_{L_{max}\_side}) \end{array} \end{array} \right] \end{aligned}$$

**Figure 16:**
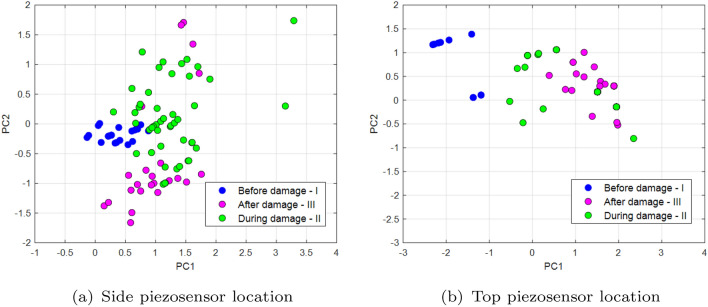
PCA diagrams calculated by supplying the propulsion system of PWM signal with 35% duty cycle from (**a**) the piezosensor located in the plane *Y–Z*, (**b**) the piezosensor located in the plane *X–Z*.

**Figure 17:**
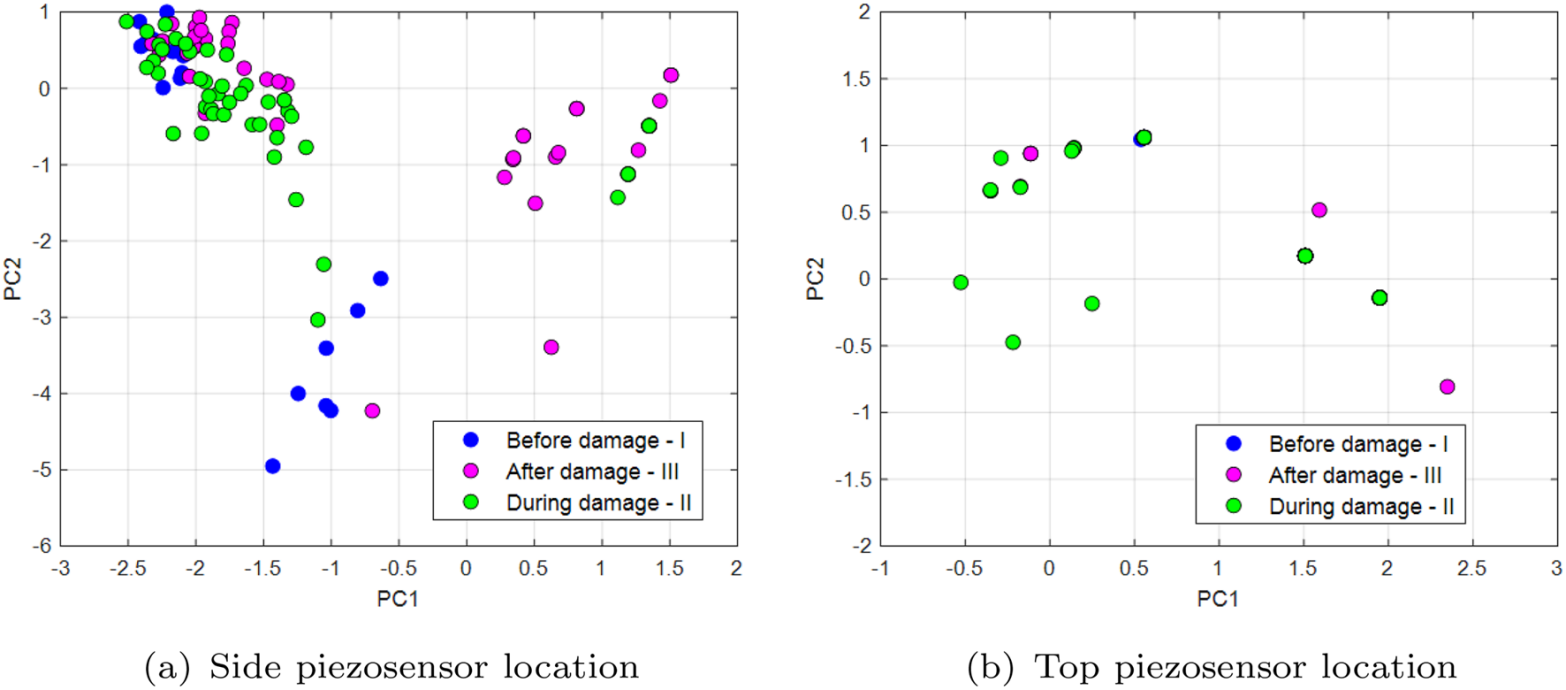
PCA diagrams calculated by supplying the propulsion system of PWM signal with 40% duty cycle from (**a**) the piezosensor located in the plane *Y–Z*, (**b**) the piezosensor located in the plane *X–Z*.

**Figure 18:**
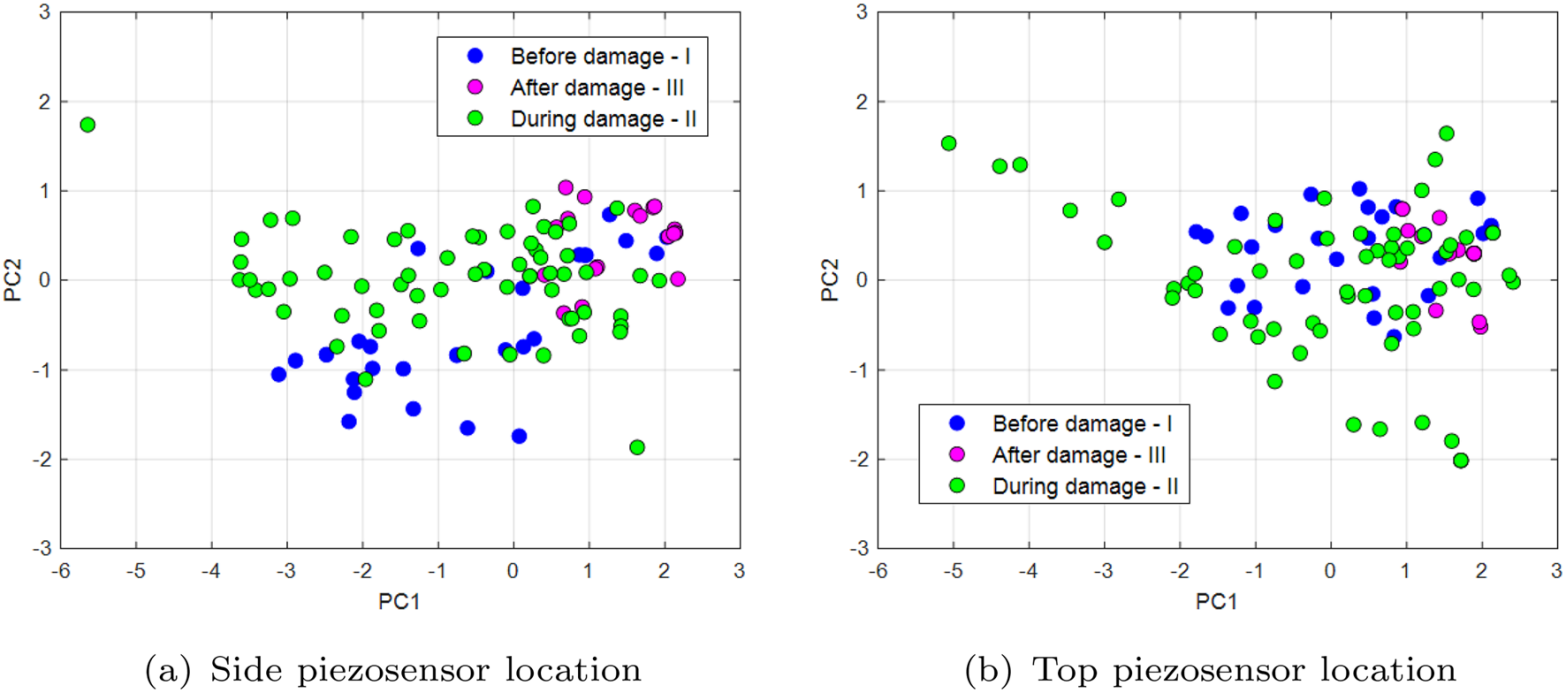
PCA diagrams calculated by supplying the propulsion system of PWM signal with 50% duty cycle from (**a**) the piezosensor located in the plane *Y–Z*, (**b**) the piezosensor located in the plane *X–Z*.

Diagrams of the principal component for the lowest PWM duty cycle (35%) are shown in Fig. 16, diagrams for 40% in Fig. 17, and the PCA diagram for the system supplied with a PWM of 50% in Fig. 18. Similar, to what was in Figs. 12 and 13, also in this case PCA diagrams calculated basing on signals recorded by the side piezosensor *Y*–*Z* are located on the left side of these figures, while those calculated based on a signal from the top piezosensor *X*–*Z*—on the right side. Also, again three different time periods *I, II* and *III* are defined that correspond to the intact propulsion system, damaged propulsion system, and the intact UAV after temporary damage.

**Figure 19:**
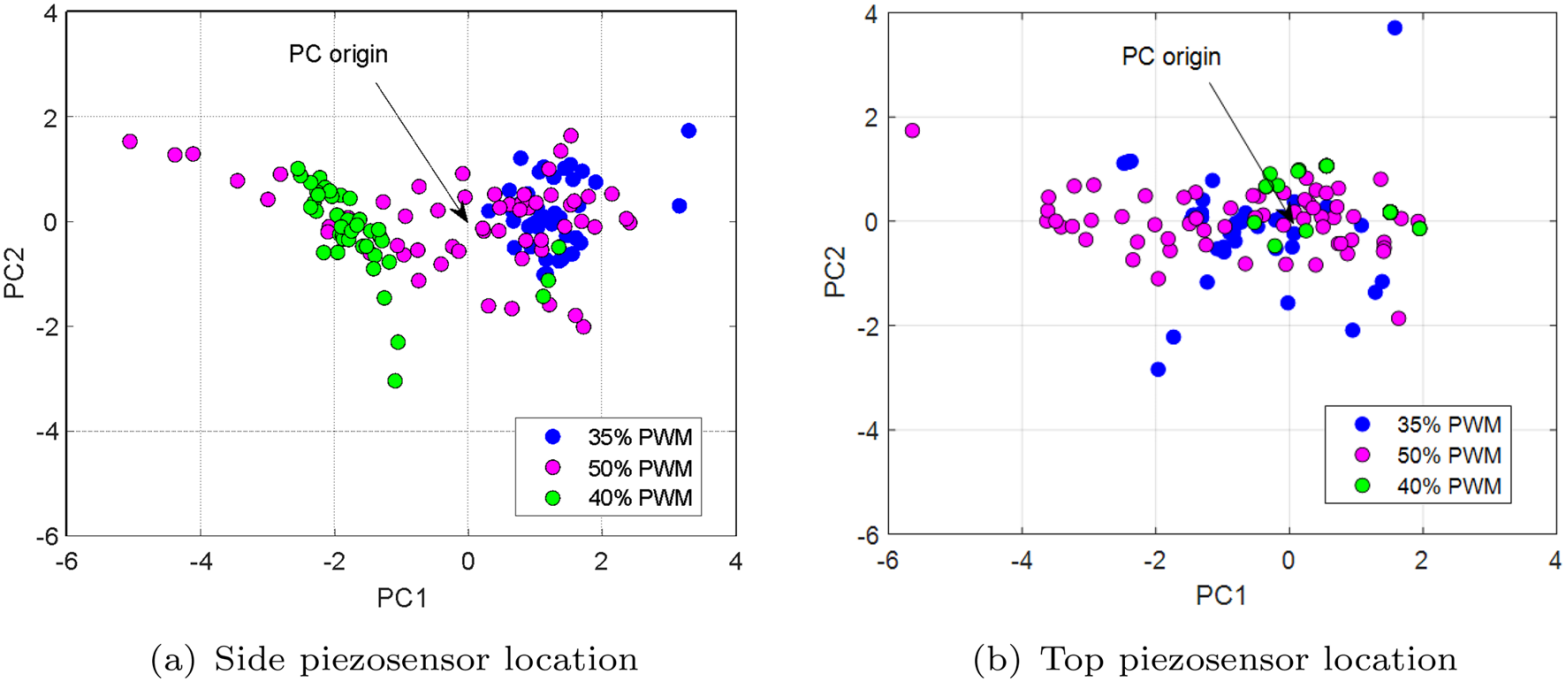
The comparison of the principal component corresponding to voltages generated by (**a**) the piezosensor located in the plane *Y–Z* (side), (**b**) the piezosensor located in the plane *X–Z* (top) for various values of duty cycle PWM signal during damage propulsion system.

The analysis of the PCA diagrams presented in Figs. 16, 17, and 18 as well as their comparison shown in Fig. 19 indicates that the monitoring of the structure of the UAV arm using the proposed method gives the expected results. Observing these diagrams can show that fault damage to the propulsion system is better detected by the side piezosensor positioned in the plane *Y*–*Z*. It is shown especially in Fig. 16a for Section *II* (35% duty cycle of PWM) where the damage to the propulsion system leads to the generation of more distribution points compared to the origin of PC in comparison to those shown in Fig. 16b—Section *II*. A similar effect can be observed in Figs. 17 and 18 Then, increasing the duty cycle of the PWM signal to 50% leads to a significant increase of the number of points on the diagrams, higher distributions of these points on the diagrams versus PC origin as well as the higher concentration of these points located farther away from PC origin (see Fig. 18b—Section *II*).

Completely different effects can be shown during the analysis of Section *II* (during damage) in Fig. 16b for the piezosensor located on the top of the UAV arm structure (plane *X*–*Z*). Then, frequency vibration of this structure close to natural frequency leads to high repeatability of voltage signals recorded by this piezosensor and consequently to decrease of the points generated on the PC that are concentrated very close to PC origin. In the case of the diagram analysis presented in Fig. 16a—Section *II* it can be shown that the chaotic vibrations of the structure are measured mainly by the side piezosensor. As a result, it leads to obtaining a larger amount of points on this diagram that in this case are located far away from the Principal Component origin.

In summary, it can be concluded that analysis of principal component diagrams as well as analysis of the size of distribution of points on the PC diagram, especially those placed further away versus PC origin, can be an additional indicator to assess fault propulsion systems located on the UAV platform (see Fig. 19). Again, it indicates, as it was similar in previous sections, that piezosensors located in the plane *Y*–*Z* (side plane of the UAV arm structure) faster detect the propulsion system fault. In addition, the obtained results allow us to determine in the nearest models by using learning machine techniques and next implementing and testing during indoor and outdoor tests.

## Conclusion

In the present paper, the diagnosis of motor damage was considered based on perturbations in the voltage changes signals obtained from piezosensors. Based on the time series of voltage changes generated by piezosensors, the methods of nonlinear analysis: Recurrence plots, recurrence quantification analysis, and principal component analysis were used. It was shown that just an investigation of the recurrence plots and recurrence quantificators helps us in the initial diagnosis of the correctness of the motor’s operation. Much better results are obtained on the basis of PCA analysis and PCA plots. The arrangement of points near the principal component origin of the coordinate system on the PCA graphs suggests that the motor is working properly. The appearance of remote points (from the coordinate system) suggests damage to the motor. The proposed methodology of investigation of the correctness of the motor’s operation also allows us to find when the frequency of vibration caused by motor operation becomes close to the resonant frequency of arm construction. It was shown that when the frequency of vibration caused by motor operation is close to the resonant frequency of arm construction, vibrations in a plane coinciding with the top of the arm become repetitive, and vibration in a plane coinciding with the side plane becomes chaotic. After finding the most optimal position of the piezoelement, the next stage of the research will be the application of a piezosensor in each arm of the UAV, to estimate the differences between them.

## Data Availability

Data will be made available on request. Correspondence and requests for materials should be addressed to A. Koszewnik.

## References

[1] Jice Z, Wu Z, Todd MD, Zhen H (2023). Bayes risk-based mission planning of unmanned aerial vehicles for autonomous damage inspection. Mech. Syst. Signal Process..

[2] Ahmadi K, Asadi D, Nabavi-Chashmi S-Y, Tutsoy O (2023). Modified adaptive discrete-time incremental nonlinear dynamic inversion control for quad-rotors in the presence of motor faults. Mech. Syst. Signal Process..

[3] Lin H-Y, Zhan J-R (2023). GNSS-denied UAV indoor navigation with UWB incorporated visual inertial odometry. Meas. J. Int. Meas. Confed..

[4] Xian B, Gu X, Pan X (2022). Data driven adaptive robust attitude control for a small size unmanned helicopter. Mech. Syst. Signal Process..

[5] Lei X, Wang R, Fu F (2022). An adaptive method of attitude and position estimation during GPS outages. Meas. J. Int. Meas. Confed..

[6] Boursianis AD, Papadopoulou MS, Diamantoulakis P, Liopa-Tsakalidi A, Barouchas P, Salahas G, Karagiannidis G, Wan S, Goudos SK (2022). Internet of Things (IoT) and agricultural unmanned aerial vehicles (UAVs) in smart farming: A comprehensive review. Internet Things.

[7] Sahoo SK, Mudligiriyappa N, Algethami AA, Manoharan P, Hamdi M, Raahemifar K (2022). Intelligent trust-based utility and reusability model: enhanced security using unmanned aerial vehicles on sensor nodes. Appl. Sci..

[8] Zheng X, Li H, Ahn CK, Yao D (2022). NN-based fixed-time attitude tracking control for multiple unmanned aerial vehicles with nonlinear faults. IEEE Trans. Aerosp. Electron. Syst..

[9] Xu Y, Weng X, Zhang J (2022). Real-time parameter identification method for a novel blended-wing-body tiltrotor UAV. Meas. J. Int. Meas. Confed..

[10] Mitronikas, E., Papathanasopoulos, D., Athanasiou, G., & Tsotoulidis, S. Hall-effect sensor fault identification in brushless DC motor drives using wavelets. In *Proceedings of the 2017 IEEE 11th International Symposium on Diagnostics for Electrical Machines, Power Electronics and Drives, SDEMPED 2017*, 131057. 10.1109/DEMPED.2017.8062391 (2017)

[11] Ciaburro G, Iannace G (2020). Improving smart cities safety using sound events detection based on deep neural network algorithms. Informatics.

[12] Faiz, J. & Ahmad, J. Interturn fault diagnosis in brushless direct current motors—A review. In *Proceedings of the IEEE International Conference on Industrial Technology.*10.1109/ICIT.2018.8352217 (2018).

[13] Medeiros RL, Filho AC, Ramos JG, Nascimento TP, Brito AV (2019). A novel approach for speed and failure detection in brushless DC motors based on chaos. IEEE Trans. Ind. Electron..

[14] Veras FC, Lima TLV, Souza JS, Ramos JG, Lima Filho AC, Brito AV (2019). Eccentricity failure detection of brushless DC motors from sound signals based on density of maxima. IEEE Access.

[15] Ghalamchi B, Jia Z, Mueller MW (2020). Real-time vibration-based propeller fault diagnosis for multicopters. IEEE/ASME Trans. Mechatron..

[16] Ambroziak L, Ołdziej D, Koszewnik A (2023). Multirotor motor failure detection with piezo sensor. Sensors.

[17] Cabahug James, Eslamiat Hossein (2022). Failure detection in quadcopter UAVs using K-means clustering. Sensors.

[18] Benini, A., Ferracuti, F., Monteriu, A., & Radensleben, S. Fault detection of a UAV using acceleration measurements. In: *18th European Control Conference, ECC 2019*, pp. 3990-3995, 8796198. 10.23919/ECC.2019.8796198 (2019)

[19] Qi, X., Theilliol, D., Qi, J., Zhang, Y., Han, J., & Song, D. Fault diagnosis and fault tolerant control methods for manned and unmanned helicopters: A literature review. In *Proceedings of the 2013 Conference on Control and Fault-Tolerant Systems (SysTol)* 132–139 (2013).

[20] Bondyra, A., Gasior, P., Gardecki, S., & Kasiński, A. Fault diagnosis and condition monitoring of UAV rotor using signal processing. In *2017 Signal Processing: Algorithms, Architectures, Arrangements, and Applications (SPA)* 233–238. 10.23919/SPA.2017.8166870 (2017).

[21] Fu, J., Sun, C., Yu, Z., & Liu, L. A hybrid CNN-LSTM model based actuator fault diagnosis for six-rotor UAVs. In *Proceedings of the 31st Chinese Control and Decision Conference, CCDC 2019* 410–414, 8832706, 10.1109/CCDC.2019.8832706 (2019)

[22] Cheng D-L, Lai W-H (2019). Application of self-organizing map on flight data analysis for quadcopter health diagnosis system. Int. Arch. Photogramm. Remote Sens. Spat. Inf. Sci..

[23] Tong Z, Chen Z, Zhu C (2022). Nonlinear dynamics analysis of cryptocurrency price fluctuations based on Bitcoin. Finance Res. Lett..

[24] Majumdar D, Bose C, Sarkar S (2022). Transition boundaries and an order-to-chaos map for the flow field past a flapping foil. J. Fluid Mech..

[25] Dzienis P, Zaborowska I, Mosdorf R (2022). JRP analysis of synchronization loss between signals recording during bubble departures. Nonlinear Dyn..

[26] Ghouli Z, Litak G (2023). Effect of high-frequency excitation on a bistable energy harvesting system. J. Vib. Eng. Technol..

[27] Ambrożkiewicz B, Syta A, Gassner A, Georgiadis A, Litak G, Meier N (2022). The influence of the radial internal clearance on the dynamic response of self-aligning ball bearings. Mech. Syst. Signal Process..

[28] Perez M, Billon K, Gerges T, Cabsal J-F, Cabrera M, Chesne S, Jean-Mistral C (2022). Vibration energy harvesting on a drone quadcopter based on piezoelectric structures. Mech. Ind..

[29] Koszewnik A, Ołdziej D (2019). Performance assessment of an energy harvesting system located on a copter. Eur. Phys. J. Spec. Top..

[30] Koszewnik A, Leśniewski K, Pakrashi V (2021). Numerical analysis and experimental verification of damage identification metrics for smart beam with MFC elements to support structural health monitoring. Sensors.

[31] Eckmann JP, Oliffson Kamphorst O, Ruelle D (1987). Recurrence plots of dynamical systems. Europhys. Lett..

[32] Fraser AM, Swinney HL (1986). Independent coordinates for strange attractors from mutual information. Phys. Rev. A.

[33] Liebert W, Schuster HG (1989). Proper choice of the time delay for the analysis of chaotic time series. Phys. Lett. A.

[34] Marwan N, Carmen Romano M, Thiel M, Kurths J (2007). Recurrence plots for the analysis of complex systems. Phys. Rep..

[35] Mindlin GM, Gilmore R (1992). Topological analysis and synthesis of chaotic time series. Phys. D.

[36] Zbilut JP, Zaldivar-Comenges JM, Strozzi F (2002). Recurrence quantification based Liapunov exponents for monitoring divergence in experimental data. Phys. Lett. Sect. A.

[37] Zbilut JP, Webber CL (1992). Embeddings and delays as derived from quantification of recurrence plots. Phys. Lett. A.

[38] Marwan N, Wessel N, Meyerfeldt U, Schirdewan A, Kurths J (2002). Recurrence-plot-based measures of complexity and their application to heart-rate-variability data. Phys. Rev. E.

[39] Pearson K (1901). On lines and planes of closest fit to systems of points in space. Lond. Edinb. Dublin Philos. Mag. J. Sci..

